# New Latency Reversing Agents for HIV-1 Cure: Insights from Nonhuman Primate Models

**DOI:** 10.3390/v13081560

**Published:** 2021-08-06

**Authors:** Katherine M. Bricker, Ann Chahroudi, Maud Mavigner

**Affiliations:** 1 Department of Pediatrics, Emory University School of Medicine, Atlanta, GA 30322, USA; kmbrick@emory.edu (K.M.B.); ann.m.chahroudi@emory.edu (A.C.); 2 Yerkes National Primate Research Center, Emory University, Atlanta, GA 30329, USA; 3 Emory + Children’s Center for Childhood Infections and Vaccines, Atlanta, GA 30322, USA

**Keywords:** HIV-1 cure, shock and kill, latency reversing agents, nonhuman primates

## Abstract

Antiretroviral therapy (ART) controls human immunodeficiency virus 1 (HIV-1) replication and prevents disease progression but does not eradicate HIV-1. The persistence of a reservoir of latently infected cells represents the main barrier to a cure. “Shock and kill” is a promising strategy involving latency reversing agents (LRAs) to reactivate HIV-1 from latently infected cells, thus exposing the infected cells to killing by the immune system or clearance agents. Here, we review advances to the “shock and kill” strategy made through the nonhuman primate (NHP) model, highlighting recently identified latency reversing agents and approaches such as mimetics of the second mitochondrial activator of caspase (SMACm), experimental CD8+ T cell depletion, immune checkpoint blockade (ICI), and toll-like receptor (TLR) agonists. We also discuss the advantages and limits of the NHP model for HIV cure research and methods developed to evaluate the efficacy of in vivo treatment with LRAs in NHPs.

## 1. Introduction

Over 37 million individuals are living with human immunodeficiency virus (HIV-1) worldwide [1]. Highly active antiretroviral therapy (ART) reduces HIV-1 plasma viral loads below detectable limits and, thus, substantially reduces transmission as well as mortality and morbidity associated with HIV-1 infection. However, ART does not eradicate HIV-1 as the virus persists in a long-lived reservoir of latently infected cells that can seed viral rebound if ART is interrupted [2,3,4]. ART accessibility remains limited in low-income countries and for those who have access to ART, treatment can be associated with stigma, long-term toxicity, and an inordinate economic burden for individuals and public health systems.

Developing approaches to eliminate or reduce the viral reservoir that could lead to a cure or lifelong remission of HIV-1 infection thus remains a key priority in HIV-1/AIDS research. Among the strategies being pursued toward eliminating the latent reservoir, the “shock and kill” approach aims to induce HIV-1 expression from latently infected cells using latency reversal agents (LRAs) in order to facilitate the clearance of these cells by the host immune response or by administered clearance agents, with the ultimate goal of reducing the size of the viral reservoir [5,6]. Evaluating putative LRAs and identifying the best strategies to reactivate the latent viral reservoir in a preclinical setting, such as the nonhuman (NHP) model, are essential to HIV-1 cure efforts. Here, we review recent advances in the “shock and kill” approach, with a focus on latency reversal in NHP models.

## 2. Nonhuman Primates for HIV-1 Cure Research

NHPs have long been established as robust animal models of HIV-1 infection, revealing critical aspects of HIV-1 immunopathogenesis and providing outstanding platforms for vaccine research. Infection of Asian monkeys such as rhesus (*Macaca mulatta*), cynomolgus (*Macaca fascicularis*) and pigtailed (*Macaca nemestrina*) macaques with simian immunodeficiency virus (SIV) or simian/human immunodeficiency virus (SHIV) approximates critical aspects of HIV-1 immunopathogenesis, including acute infection events and disease progression [7,8]. With the advent of potent ART regimens able to suppress SIV replication to undetectable levels as observed in people living with HIV-1, NHPs now also represent excellent models to study HIV-1 persistence and evaluate curative therapeutic strategies. In this section, we discuss the advantages and limitations of the NHP models for HIV-1 cure research as well as the similarities and differences in terms of viral reservoir between HIV-1 and SIV pathogenic infections.

ART can suppress SIV and SHIV replication in macaques to levels below the limit of detection of standard viral load assays [9,10]. A few studies have reported low (<100 copies/mL), but persistent, levels of SIV RNA in the cerebrospinal fluid of long-term ART-suppressed SIV-infected macaques comparable to those observed in people living with HIV-1 on suppressive ART [11,12,13,14]. Similar to HIV-1, SIV is able to persist in an infected macaque despite ART, as shown by a rapid rebound of viral RNA in plasma within 7 to 21 days when ART is interrupted, even following early administration of ART [15]. HIV-1 and SIV persist on ART in a long-lasting reservoir of latently infected CD4+ T cells and other non-lymphocyte populations such as macrophages [16,17].

Key properties of the HIV-1 reservoir have also been observed in SIV infection, including a very early establishment of the reservoir, its anatomical and cellular distribution, and dynamics over time. The HIV-1 reservoir is remarkably stable on ART, with waxing and waning of T cell clones contributing to this overall stability [4,18,19]. The half-life of latently infected cells capable of producing replication-competent virus is estimated to be 44 months [4]. An early study using mathematical modeling showed that ART-induced suppression of viremia in pigtailed macaques was biphasic with half-lives similar to what is observed in HIV-1 patients treated with ART [20]. The anatomical distribution of CD4+ T cells harboring viral DNA has been extensively evaluated in the NHP model to an extent not possible in humans living with HIV-1. North et al. demonstrated that viral DNA and RNA levels in RT-SHIV-infected, ART-treated macaques were highest in lymphoid tissues including the spleen, lymph nodes, and gastrointestinal (GI) tract [21]. These results have been confirmed in later studies with more potent ART regimens and longer periods of treatment [22,23]. The central nervous system has also been implicated as a site of viral persistence in NHP models potentially due to poor ART penetrance [24]. The anatomic distribution of the viral reservoir has been described in rhesus macaque infants using pediatric models of postnatal oral SIV/SHIV transmission and ART treatment. These studies showed that viral RNA and DNA decreased on ART in lymphoid tissues but remained stable in the CNS of ART-suppressed rhesus macaque infants and identified the GI tract as a site of persistent low-level viral transcription during ART [25,26]. This model now allows the testing of potential HIV-1 cure strategies specifically in a pediatric setting that is difficult to assess in humans [27].

Through the NHP model, it has been established that the viral reservoir is seeded early in acute infection [28]. Studies of early ART initiation in rhesus macaques infected intrarectally (i.r.) with SIVmac251 showed a rebound of plasma viremia following analytical treatment interruption (ATI) in all animals when treatment was initiated past 2 days post-infection [15,29]. In a follow up study in rhesus macaques challenged intravenous (i.v.) with SIVmac239X, a majority but not all of the animals treated with ART between day 4 and 6 post-infection also experienced rebound viremia after ATI [30]. Similarly, viral rebound following ATI was observed in HIV-1-infected individuals who were initiated on ART very early following infection, at the transition between the “eclipse phase” of HIV-1 infection, during which HIV is undetectable, and Fiebig stage I, during which HIV-1 RNA can be detected in the blood but not HIV antigens or antibodies [31,32]. Additionally, it has been known for decades that a large majority of the latent HIV-1 reservoir is comprised of defective proviruses, and a recent study demonstrated that defective viral genomes also predominated in SIV infection under ART [33]. Finally, both HIV-1 and SIV share similarities in the cellular composition of the reservoir with viral persistence in all subsets of memory CD4+ T cells, and high levels of viral DNA found in central and transitional memory CD4+ T cells [25,34]. Viral DNA has also been detected in the more recently identified stem cell memory CD4+ T (SCM) cells in both humans and NHPs on suppressive ART, with an increased contribution of the SCM to the reservoir on ART over time [35,36,37]. Finally, persistent HIV-1 and SIV/SHIV DNA is also found in naïve CD4+ T cells that might contribute to the reservoir to higher levels than initially thought [25,26,38].

However, NHPs do not fully recapitulate HIV-1 persistence in humans, notably because of the logistical and financial barriers to treating macaques with ART for many years to decades, as people living with HIV-1 are, and because of differences between SIV/SHIV and HIV-1. Significant differences are seen in the SIV viral envelope (Env) preventing the use of SIV/NHP models to test HIV-1 Env-directed clearance agents, such as broadly neutralizing antibodies, in the setting of “shock and kill” approaches. To overcome this limitation, SHIVs expressing HIV-1 Env glycoproteins have been engineered and have been optimized over time to increase pathogenicity and better reflect HIV-1 infection notably in terms of reservoir formation [39,40,41,42]. Characterization of persisting proviruses in ART-suppressed rhesus macaques also showed several differences in terms of sequence evolution as compared to HIV-1, including the detection of more frequent intact proviruses, a larger fraction of G-to-A hypermutations, and less frequent large internal deletions [33]. Another key difference was the limited number of identical SIV sequences although an earlier study using integration site analysis suggested similar clonal expansion of latently infected cells in macaques and humans [43]. The differences in viral sequence evolution between SIV and HIV can be attributed to the host biology, viral genetics, and/or the timescale of infection and ART treatment.

**Figure 1:**
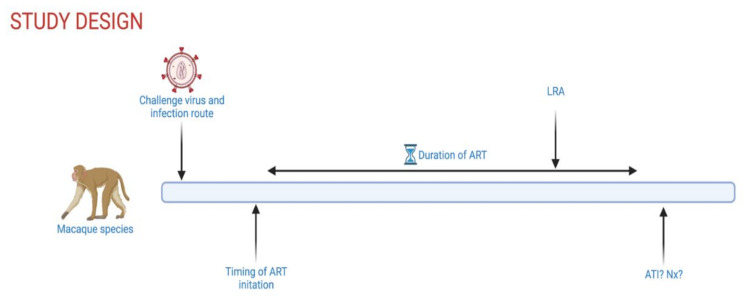
Schematic overview of controllable experimental parameters in NHP HIV-1 cure studies. ART, antiretroviral therapy; LRA, latency reversing agent; ATI, analytical treatment interruption; Nx, elective necropsy. Created with Biorender.com.

Additionally, several parameters have to be considered when designing a study testing “shock and kill” approaches in NHPs (Figure 1), including viral strains, infection dose, infection route, time of ART initiation, ART duration, and study endpoint. SIVmac239, a molecular clone, and SIVmac251, a viral swarm with significant genetic heterogeneity, are the two most commonly used strains, and reservoir establishment and maintenance have been demonstrated with both strains. The use of SHIVs allows for testing of interventions that target the human Env protein but were historically less pathogenic, with more frequent spontaneous control of viremia than during SIV infection. More recently engineered SHIVs that contain primary Envs with residue substitutions at Env375 demonstrated improved viral kinetics and now represent a better model of HIV latency [44]. The intravenous route is often used to ensure successful infection in one challenge, although mucosal challenge (intrarectal, intravaginal, oral) is likely to more closely mimic early infection events seen in most HIV-1 transmission in humans. While it is known that timing of ART initiation and ART duration quantitatively and qualitatively affect the viral reservoir, studies comparing the impact of viral strains or infection route on the viral reservoir are limited. Ultimately, study design parameters best suited to test a specific intervention are selected while compromising with time constraints dictated by the use of animal models. Further, in humans, MHC class I alleles are inherited with little crossover. In contrast, rhesus macaques have significantly more haplotype diversity as several MHC class I alleles exist on each chromosome with higher potential for crossover [45,46]. This variability likely impacts CD8+ T cell function, which may influence the “kill” arm in a “shock and kill” cure strategy. Nevertheless, the NHP model of SIV infection represents a robust model to test safety and efficacy of curative therapeutics in a controlled manner not possible in clinical trials. Additionally, the NHP model offers specific powerful tools to study viral latency and reactivation in relevant anatomical sites, such as the use of genetically barcoded viruses to monitor viral variants as well as in vivo and in situ viral detection methods that are, for the most part, not plausible to use in humans (detailed in the next section). The NHP model also allows for extensive longitudinal tissue sampling, ATI, and elective necropsies with the collection of a plethora of tissues, thus permitting an in vivo, in-depth anatomical site-driven evaluation of novel strategies aimed at eliminating or reducing the viral reservoir.

## 3. Latency Reversing Agents in NHP Studies

LRAs have been extensively reviewed elsewhere [47,48]; here, we focus on the most recent LRAs tested in rhesus macaques and their potential applicability to human trials. The term latency reversal here is used to describe the induction of HIV-1 expression (RNA in cells or plasma) above that of controls or pre-LRA treatment levels. We use “on-ART viremia” to describe the ability of a given LRA to stimulate virus production from infected cells that is measurable above the limit of detection of standard plasma viral load assays while daily ART is maintained.

### 3.1. SMAC Mimetics

NF-kB signaling plays an important role in HIV-1 transcription and has thus been the target of several LRA candidates, notably PKC agonists [49]. However, the canonical pathway is fairly non-specific and induces broad systemic inflammation, leading to cellular cytotoxicity [49]. Targeting the non-canonical NF-kB pathway with mimetics of the second mitochondrial activator of caspase (SMACm) represents a more selective approach, and our groups have identified the SMACm AZD5582 as a potent in vivo LRA in NHPs (Table 1). Treatment with AZD5582 induced on-ART viremia in 5/9 (55%) ART-suppressed SIVmac239-infected rhesus macaques that received ten doses of AZD5582 with 46% of total viral loads in these 5 macaques above the limit of detection over the treatment period [50]. It is not completely clear why viral reactivation was not induced in 100% of the animals treated with AZD5582, but we note that macaques without evidence of latency reversal during treatment had reduced viral replication pre-ART and lower SIV DNA levels in peripheral CD4+ T cells before intervention, implicating reservoir size as a contributing factor. Nevertheless, in the majority of animals, treatment with AZD5582 induced multiple instances of sustained on-ART viremia, reaching levels as high as 1390 copies/mL. Additionally, cell-associated SIV RNA levels in resting CD4+ T cells isolated from lymph nodes were significantly higher in animals who received ten doses of AZD5582 as compared to ART-only controls. Of note, AZD5582 treatment also resulted in the induction of HIV-1 RNA expression in the blood and tissues of ART-suppressed bone marrow/liver/thymus humanized mice.

In subsequent studies, we administered the same dose and regimen of AZD5582 to 8 ART-suppressed SIVmac251-infected rhesus macaque infants. In this model, on-ART viremia was observed in 5/8 infant macaques (62.5%) in response to AZD5582 treatment. The peak on-ART viremia and the frequency of positive viral loads in responding animals were lower in infant than in adult rhesus macaques, and we are actively investigating the potential mechanisms for this difference [51]. However, this study shows that targeting the non-canonical NF-kB pathway can also induce latency reversal in macaque infants. Further work evaluating a combination of AZD5582 with bispecific HIVxCD3 retargeting molecules (DART) in 8 SHIV.C.CH505-infected rhesus macaques did not reveal on-ART viremia following treatment [52], with the corresponding virologic data again suggesting a correlation between the latency-reversing activity of AZD5582 and viral reservoir size before intervention. Finally, as detailed below, the combination of AZD5582 and CD8+ T cell depletion resulted in latency reversal in 100% of SIV-infected ART-suppressed rhesus macaques [53]. Altogether, these results from several independent studies show that the SMACm AZD5582 efficiently reverses HIV-1/SIV latency in vivo via the selective activation of the non-canonical NF-kB pathway.

### 3.2. CD8 Depletion

A role of CD8+ T cells in controlling virus transcription during ART was recently described, notably through in vivo experimental cell depletions in NHPs using CD8-specific monoclonal antibodies (mAbs) (Table 2) [53,54,55,56,57,58]. Studies in ART-suppressed SIV-infected rhesus macaques have demonstrated that experimental antibody-mediated CD8α+ cell depletion is consistently followed by high level viremia while ART is maintained [54,57]. A striking inverse correlation is observed between the level of circulating CD8+ T cells and plasma viral loads, with the on-ART viremia seen during CD8 depletion subsiding when these cells are replenished. These results show that experimental CD8α+ cell depletion disrupts SIV latency, thus acting as a LRA [54]. We note that CD8α-depleting antibody depletes not only CD8+ T cells but also natural killer cells (NK), as the majority of circulating NK cells in macaques express CD8α on their cell surface [59]. Alternatively, the CD8β-depleting antibody selectively depletes CD8+ T cells, although less efficiently than CD8α depletion [58,60]. In rhesus macaques infected with the barcoded virus SIVmac239M, a more modest effect of CD8β depletion on virus reactivation was shown, with no significant difference in on-ART viremia episodes between CD8β-depleted RM and ART-only controls [61]. However, these results also implicated CD8+ T cells in virus suppression during ART interruption, with a 2-log increase in post-ART plasma viremia in effectively CD8+ T cell-depleted rhesus macaques as compared to controls.

Removal of CD8+ T cells allowed treatment with the IL-15 superagonist N-803, that did not reverse latency by itself, to result in robust and prolonged virus reactivation in ART-suppressed rhesus macaques infected with SIVmac239 or SHIVSF162P3 [57]. Specifically, in 21 SIVmac239-infected rhesus macaques on suppressive ART for over a year, treatment with N-803 only resulted in on-ART viremia above baseline when CD8+ cells were depleted using the anti-CD8α mAb MT807R1 (14/14 with the combination and 0/7 with N-803 alone). Similar results were obtained in a smaller pilot study in which N-803 and MT807R1 administration was performed in 5 ART-suppressed SHIVSF162P3-infected rhesus macaques. Although not as potent as the CD8α-depleting antibody, on-ART viral reactivation was also observed in 3 out of 5 SHIVSF162P3-infected rhesus macaques when the CD8β-depleting antibody, CD8b255R1, was used in combination with N-803 following one year of daily ART [58]. Additionally, CD8α depletion also enhanced the latency reversal activity of the SMACm AZD5582 in ART-suppressed SIV-infected macaques [53], with 100% of animals treated with MT807R1 and AZD5582 experiencing on-ART viremia versus 56% treated with AZD5582 only and 57% treated with MT807R1 only. Furthermore, the frequency of viremic episodes and the level of on-ART viremia during the treatment period was greater in the combined CD8 depletion and AZD5582 group as compared to the others. Removal of CD8+ cells has thus been shown in independent studies to potentiate viral reactivation for two different LRA compounds. Understanding the mechanism(s) by which CD8+ T cells suppress HIV/SIV transcription could be key to developing improved LRAs and novel approaches to an HIV cure.

### 3.3. Immune Checkpoint Inhibitors

Persistent antigen exposure during chronic HIV-1/SIV infection results in reduced effector function of CD8+ T cells, or immune exhaustion, characterized by the overexpression of immune checkpoint receptors such as PD-1, LAG-3, TIM-3, TIGIT, and CTLA-4 [62]. This exhaustion can be partially restored with the use of immune checkpoint blockade using monoclonal antibodies targeting the cell surface receptors listed above [63]. Immune checkpoint inhibitors (ICIs) also may play a role in reactivating the viral reservoir as they can bind to cell surface markers on latently infected CD4+ T cells, preventing the inhibitory signals and ultimately triggering viral transcription [64]. Administration of two immune checkpoint blockade antibodies against CTLA-4 and PD-1 in ART-suppressed SIVmac239-infected rhesus macaques resulted in on-ART viremia in 2/6 (33%) of CTLA-4-treated, 2/6 (33%) of PD-1-treated, and 4/7 (57%) of CTLA-4/PD-1-combination-treated macaques with 9/28 (32%) of total viral loads in responding animals above the limit of detection throughout combination treatment [65]. Alternatively, in a study combining GS-9620 with a chimeric human–rhesus version of the anti-PD-1 antibody nivolumab in SIVmac251-infected rhesus macaques, no changes in on-ART viremia or viral rebound kinetics during ATI was observed, although ART was initiated 7 days post-infection [66] (Table 3).

### 3.4. TLR Agonists

Toll-like receptors (TLRs) are a family of pattern recognition receptors playing a critical role in the innate immune system by detecting pathogen-associated molecular patterns. TLR agonists have drawn interest in the HIV-1 cure field due to their potential to both reactivate the latent reservoir and to modulate the immune system [67]. Among them, TLR-7 agonists have been extensively tested in macaques. In a first study, transient viremia was observed following repeated administration of GS-9620 or its tool compound GS-986 in all 19 SIVmac251-infected rhesus macaques that had been virally suppressed for approximately 400 days. A subset of treated animals also showed a delay or absence of rebound following ART interruption [68], with 2 of 9 animals remaining aviremic for over two years. However, further studies using these TLR-7 agonists alone or in combination with other experimental agents did not demonstrate latency reversal and variable effects on the viral reservoir size were observed. In four rhesus macaques infected with SIVmac239X—a molecularly tagged synthetic swarm virus [69]—that received two rounds of GS-9620 at two-week intervals (12 doses in round 1 and 5 doses in round 2), no latency reversal was observed and GS-9620 did not influence the levels of viral DNA in PBMCs or tissues following treatment [70]. The cause of the discrepancies between these studies is unclear. Variable parameters included the virus used, the route of infection, the duration of ART, and the timing of ART initiation (see Table 4).

Two studies, led by Borducchi et. al., combined a TLR-7 agonist with either Ad26/MVA therapeutic vaccination or the broadly neutralizing antibody PGT121 in SIVmac251- or SHIV-SF162P3-infected rhesus macaques, respectively [71,72]. In both studies, animals began daily ART at 7 days post-infection and combination treatment did not induce latency reversal. However, lower viral DNA in lymph nodes, delayed viral rebound following analytical treatment interruption, and lower rebound set point were observed as compared to sham controls. Our group recently performed a similar study combining the TLR-7 agonist GS-986 with Ad48/MVA therapeutic vaccination in 8 ART-treated SIVmac251-infected macaque infants [27]. We found that plasma viral loads on ART did not significantly differ between macaques treated with GS-986 and ART-only controls, indicating that GS-986 did not serve as an LRA in this setting.

## 4. Evaluating LRAs in NHPs

**Figure 2:**
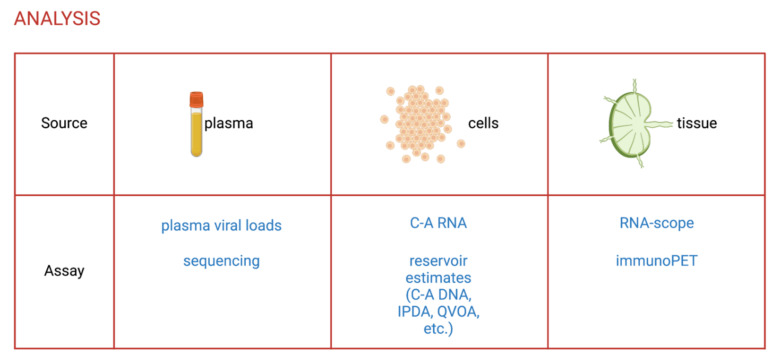
Assays to evaluate the efficacy of LRAs in NHPs. C-A, cell-associated; IPDA, intact proviral DNA assay; QVOA, quantitative viral outgrowth assay. Created with Biorender.com.

With the validation of NHP models for HIV-1 cure studies, it became essential to develop assays that accurately measure the size of the latent reservoir and viral reactivation specifically in SIV- and SHIV-infected rhesus macaques. Here, we describe some of these assays that have been adapted from HIV-1 research to the NHP model or developed to take advantage of specificities of this model (detailed in Figure 2).

### 4.1. Evaluating Latency Reversal

The most commonly used assay to evaluate viral reactivation induced by in vivo treatment with LRA candidates is the quantification of viral RNA in the plasma by reverse transcription polymerase chain reaction (RT–PCR) [73,74,75]. As control of viral replication is defined by sustained plasma viral load below the limit of detection of the assay used (generally 10 to 60 copies of SIV RNA per mL of plasma for standard assays) [9], detection of on-ART viremia above this limit is indicative of viral reactivation. Additionally, ultrasensitive assays detecting down to 1 copy of SIV RNA per mL have been developed [76]. While the clinical relevance of low-level increases in viremia is unknown, it allows a sensitive evaluation of a given LRA’s activity. Viral reactivation induced by in vivo treatment with LRAs can also be evaluated by measuring cell-associated SIV RNA by RT–PCR in CD4+ T cells isolated from peripheral blood and tissues [50,77]. Important spatial information is lost when cells are isolated from tissues and this process can lead to changes in cell phenotypes or viral expression. These limits can be overcome by using in situ or in vivo methods of viral detection, described in the next section.

### 4.2. Identifying the Source of Reactivated Virus

In addition to measuring viral reactivation following the administration of LRAs, determining the source of reactivated viruses is also an important area of investigation that could help in designing targeted cure strategies. With facilitated access to tissue samples, including repetitive tissue biopsies and extensive tissue collection at necropsy, animal models are key to investigating the anatomic and cellular origin(s) of the reactivated virus.

Among the various in situ technologies available, RNAscope has become the most-widely used method to track HIV-1 and SIV infection within anatomically intact tissue compartments [25,78,79,80,81]. This next-generation in situ hybridization method specifically and sensitively detects viral particles and cells expressing viral RNA. Cell phenotyping can be performed within the same tissue section, thus allowing for cellular and anatomical characterization of the reactivating viral reservoir. Another method using immunoPET (antibody-targeted positron emission tomography) has been developed specifically to capture total-body SIV replication, with the exception of the CNS [82]. Poly(ethylene glycol)-modified, 64Cu-labeled SIV gp120-specific antibody is administered intravenously to macaques to monitor kinetics of viral replication in tissues over time. Of note, this whole-body imaging method can be repeated within the same animal without adverse effects and does not require sacrifice of the animal. In ART-suppressed rhesus macaques, SIV was visualized by immunoPET in multiple tissues, including colon, spleen, and nasal-associated lymphoid tissue. Recently, this method was also used to study viral rebound in SHIV-infected RM infants, showing a rapid expansion of infected cells in the gastrointestinal tract after ATI [83]. SHIV Env-expressing cells were detected in tissues prior to the rebound in viremia, suggesting that ImmunoPET could be used to evaluate LRA-induced viral reactivation.

Genetically barcoded SIV strains represent another tool to investigate the origin and extent of virus reactivation. Barcoded viruses are generated by inserting a small and genetically unique tag into the viral genome, which is retained during replication, thus allowing quantification by next-generation sequencing. Keele et al. generated the barcoded SIVmac239M by inserting a 34-base genetic barcode between the Vpx and Vpr accessory genes of the well-characterized molecular clone SIVmac239 [84]. The virus stock that includes approximately 10,000 individual barcoded viral variants can be used to infect macaques and individual barcoded viral variants are then tracked during infection. During LRA treatment, barcode sequencing could be used to track SIV RNA in plasma and from specific cells and tissues. A second-generation barcoded SIVmac239 stock with an increased number of variants as well as a barcoded SHIV that can be valuable for antibody-mediated clearance of infected cells in “shock and kill” approaches have recently been generated [85].

The methods described above allow investigation into the source of viral reactivation on a level not possible in people living with HIV-1, highlighting a key contribution that the NHP model of SIV and SHIV infection can make in the quest for an HIV-1 cure.

### 4.3. Quantifying the Viral Reservoir

While latency reversal itself is unlikely to reduce the viral reservoir, estimating its size is of critical importance to evaluate combined “shock and kill” cure approaches, and several assays have been adapted to the SIV/RM model for that purpose. Quantitative PCR assays are inexpensive and simple methods estimating the viral reservoir size by measuring all or multiple different forms of viral DNA. The most commonly used PCR assay quantifies total cell-associated SIV DNA with no distinction between integrated and unintegrated DNA [50,77]. To specifically quantify integrated viral DNA in SIV/SHIV-infected rhesus macaques, our group has optimized a repetitive sampling Alu-gag PCR assay that uses a polyclonal integration standard to reflect the variability of SIV integration [86]. While clinically relevant, quantitative PCR assays largely overestimate the size of the reservoir, as they indistinctively detect intact and defective proviruses.

The gold standard assay to specifically estimate the replication-competent reservoir size has, for years, been the quantitative viral outgrowth assay (QVOA). The large cell input required for this assay typically limits its application to peripheral blood mononuclear cells collected by leukapheresis from people living with HIV-1. In NHPs, large numbers of CD4^+^ T cells can be isolated from lymphoid tissues collected at necropsy, including key reservoir compartments such as the lymph nodes or spleen, and several groups, including ours, have developed SIV QVOAs. Similar to the HIV-1 QVOA [87,88,89], the simian version uses serial dilutions of CD4^+^ T cells isolated from ART-suppressed SIV-infected rhesus macaques maintained in co-culture with feeder cells after ex vivo mitogenic stimulation [26,52,53,57,90,91,92,93,94,95,96]. The murine viral outgrowth assay (MVOA) is a variant of the traditional QVOA in which PBMCs or CD4^+^ T cells can be transferred from ART-suppressed HIV-1-infected patients or SIV-infected macaques to humanized or simianized mice, which are then monitored for viremia following CD8^+^ T cell depletion [97]. While this assay may allow for better viral amplification than cell culture, it is not quantitative. Overall, QVOAs are labor-intensive, relatively expensive, and underestimate the size of the latent reservoir because one round of activation does not induce all proviruses. Several alternatives to the QVOA have been developed and adapted to the RM model of SIV infection, including a tat/rev-induced limiting dilution assay (TILDA) that measures the frequency of cells producing viral multiply spliced Tat/Rev RNA upon maximal ex vivo stimulation, and the intact proviral DNA assay (IPDA) that measures the frequency of cells carrying intact proviruses [33,98]. Similar to the HIV-1 IPDA [33], SIV [33], and SHIV [26], IPDAs consist of multiplex droplet digital PCR (ddPCR) reactions to distinguish and separately quantify intact proviruses from defective proviruses.

In the rapidly evolving field of HIV-1 cure research, new assays to accurately quantify and characterize the viral reservoir are continuously being developed and optimized. However, to date, the ultimate in vivo assessment of the efficacy of interventions aimed at curing HIV-1, such as “shock and kill” approaches, remains ATI. ATI has been used extensively to evaluate cure-directed interventions in NHP models, and this experimental approach is likely to play a key role in determining whether novel agents merit further assessment in human trials.

## 5. General Conclusions and Perspectives

The NHP model of SIV/SHIV infection is now a robust animal model for HIV cure research that has recently led to the identification of several approaches inducing latency reversal in vivo. Some of these new generation LRAs are being evaluated in clinical trials. The safety and biological activity of the TLR-7 agonist GS-9620 was recently assessed in a phase Ib, randomized, dose-escalation study in ART-suppressed HIV-1-infected patients. While an immune enhancing effect was observed, treatment with GS-9620 did not induce on-ART viremia or impact any other virological markers as compared to individuals treated with a placebo [99]. A subsequent study in HIV-1 controllers showed a modest impact of GS-9620 on time to viral rebound post-ATI with a median delay of 0.3 weeks as compared to a placebo group [100]. The latency reversing activity of anti-PD-1 antibody treatment alone or in combination with and anti-CTLA-4 was also evaluated in clinic with a phase I trial in ART-suppressed HIV-infected individuals with cancer. Only the combined treatment showed a very modest effect on HIV transcription with a 1.44 median fold-increase in cell-associated HIV RNA within 24 h of the first dose [101]. The latency reversing activity of SMAC mimetics has yet to be evaluated in a clinical setting.

Besides the direct translation of therapeutics to humans, NHP work on latency reversal allows a better understanding of the maintenance of HIV reservoir. Indeed, while CD8+ T cell depletion is unlikely to move forward in clinics, this approach has revealed a substantial role for CD8+ T cells in inhibiting HIV transcription on ART and in suppressing the latency reversing activity of the IL-15 superagonist N-803 and the SMACm AZD5582. While depleting CD8+ T cells as part of a HIV cure strategy might be counterintuitive, as early during infection cytotoxic lymphocyte (CTL) activity results in a net reduction of the reservoir size, these depletion studies on ART suggest that CD8+ T cells paradoxically contribute to persistence of the HIV reservoir. Elucidating the precise mechanisms involved in CD8^+^ T cell control of HIV latency may be key in achieving a cure for HIV infection.

Despite these recent successes with new generation LRAs, only a fraction of the latent reservoir is reactivated, and the pre-intervention reservoir size might influence latency reversal. Potentiating latency reversal will likely require taking the heterogeneity of the viral reservoir into consideration, to optimize penetration of the LRAs in tissues and anatomical sanctuaries and to simultaneously target multiple pathways promoting HIV latency in combination approaches. Additionally, to date, none of the tested LRAs showed a significant impact on the viral reservoir size, suggesting that supplementation with a “kill” component will be needed to achieve a functional cure. Elimination of the latently infected cells could be facilitated by therapeutic vaccination or by the administration of clearance agents such as broadly neutralizing antibodies, eCD4-Ig chimeric antigen receptor T cells (CAR-T cells), or apoptosis inducers. The leading “kill” interventions should now be tested in combination with the newly identified LRAs and NHPs will be key in evaluating these combination approaches.

## Figures and Tables

**Table 1:** Recent NHP studies testing AZD5582 as an LRA. w.p.i., weeks post-infection; RM, rhesus macaque; i.v., intravenous; C-A, cell-associated; QVOA, quantitative viral outgrowth assay.

Study	LRA	Dose	Time on ART	“Kill” (If Included)	Virus (Infection Route, Dose)	Timing of ART Initiation	Reduction to Viral Reservoir (Assay)	Key Results
Nixon and Mavigner et al. [50]	AZD5582	0.1 mg/kg (i.v.), 10 weekly doses	55–67 weeks	n.a.	SIVmac239 (3000 TCID50, i.v.)	8 w.p.i.	No (C-A DNA and QVOA)	On-ART viremia in 5/9 RMs; peak viral load at 1390 copies SIV RNA/mL of plasma; 46% events of detectable viremia in animals that showed latency reversal
Bricker et al. [51]	AZD5582	0.1 mg/kg (i.v.), 10 weekly doses	>1 year	n.a.	SIVmac251 (10^5^ TCID50, oral)	4 w.p.i.	Not measured	On-ART viremia in 5/8 RMs, peak viral load at 771 copies/mL; 6% events of detectable viremia in animals that showed latency reversal
Dashti et al. [52]	AZD5582	0.1 mg/kg (i.v.), 10 weekly doses	43 weeks	Bispecific HIVxCD3 retargeting molecules (DARTs)	SHIV.C.CH505. 375H.dCT (10 ng p27, i.v.)	16 w.p.i.	No (C-A DNA or QVOA)	No on-ART viremia, significantly lower pre-ART viral loads and pre-LRA cell-associated viral DNA levels than SIV-infected RMs in Nixon and Mavigner et al.

**Table 2:** Recent NHP studies testing CD8 depletion as an LRA. d.p.i., days post-infection; RM, rhesus macaque; i.v., intravenous, s.c., subcutaneous; TCID50, 50% tissue culture infectious dose; IU, infectious units; C-A, cell-associated; QVOA, quantitative viral outgrowth assay.

Study	LRA	Dose	Time on ART	“Kill: (If Included)	Virus	Timing of ART Initiation	Reduction to Viral Reservoir (Assay)	Key Results
Cartwright et al. [54]	Anti-CD8α Ab (MT-807R1)	50 mg/kg (i.v.)	8–32 weeks	n.a.	SIVmac239 (3000 TCID50, i.v.)	56 d.p.i.	No (C-A DNA)	On-ART viremia in 7/7 RMs
McBrien et al. [57]	CD8α depletion (CD5b255R1) + N-803	CD8α Ab: 50 mg/kg (i.v.); N-803: 100 ug/kg (s.c.) 4 bi-weekly doses	>1 year	n.a.	SIVmac239 (3000 TCID50, i.v.)	56 d.p.i.	No (C-A DNA)	N-803: No on-ART viremia; CD8 depletion: on-ART viremia in 11/14 RMs, CD8 + N-803: on-ART viremia in 14/14 RMs
Okoye et al. [61]	CD8β depletion (CD5b255R1)	50 mg/kg (i.v.)	31 weeks	n.a.	SIVmac239M (200 IU, i.v.)	12 d.p.i.	Not measured	No significant difference in on-ART viremia between CD8β-depleted and control RMs
McBrien et al. [57]	CD8β depletion (CD5b255R1) + N-803	CD8β Ab: 50 mg/kg (s.c.); N-803: 100 ug/kg (i.v.), 4 weekly doses	45 weeks	n.a.	SHIVSF162P3 (1:50 dilution of 2,032 TCID50, i.r.)	84 d.p.i.	No (C-A DNA)	On-ART viremia in 3/5 RMs
Mavigner et al. [58]	Anti-CD8α Ab (MT-807R1) + AZD5582	CD8α Ab: 50 mg/kg (i.v.); AZD5582: 0.1 mg/kg (i.v.), 5 weekly doses	84–85 weeks	n.a.	SIVmac239 (3000 TCID50, i.v.)	56 d.p.i.	No (C-A DNA and QVOA)	On-ART viremia in 6/6 RMs; 96% of viral load measurements above baseline during intervention

**Table 3:** Recent NHP studies testing immune checkpoint inhibitors as an LRA. Ab, antibody; TCID50, 50% tissue culture infectious dose; i.v., intravenous; i.r., intrarectal; o.g., oral gastric; d.p.i., days post-infection; RM, rhesus macaque; C-A, cell-associated; IPDA, intact proviral DNA assay.

Study	LRA	Dose	Time on ART	“Kill” (If Included)	Virus	Timing of ART Initiation	Reduction to Viral Reservoir (Assay)	Key Results
Harper et al. [65]	anti-CTLA-4 and/or anti-PD-1 Ab	5 mg/kg	54 weeks	n.a.	SIVmac239 (200 TCID50, i.v.)	60 d.p.i.	Yes (IPDA)	On-ART viremia in 57% of combination blockade RMs 32% of events above baseline during combined intervention; 12.5% of events above baseline during anti-PD-1 intervention; 12.5% events above baseline during anti-CTLA-4
Bekerman et al. [66]	GS-9620 and/or anti-PD-1 Ab	GS-9620: 0.15 mg/kg (o.g.), 10 bi-weekly doses; PD-1 Ab: 10 mg/kg (i.v.) 4 bi-weekly doses	>2 years	n.a.	SIVmac251 (500 TCID50, i.r.)	7 d.p.i.	No (C-A DNA and IPDA)	No on-ART viremia

**Table 4:** Recent NHP studies testing TLR agonists as an LRA. TLR, Toll-like receptor, o.g., oral gastric; AID50, 50% animal infectious dose; i.r., intrarectal; IU, infectious units; i.v., intravenous; TCID50, 50% tissue culture infectious dose; d.p.i., days post-infection; RM, rhesus macaque; C-A, cell-associated.

Study	LRA	Dose	Time on ART	“Kill” (If Included)	Virus	Timing of ART Initiation	Reduction to Viral Reservoir (Assay)	Key Results
Lim et al. [67]	GS-986	Dose escalation 0.1 mg, 0.2 mg, and 0.3 mg/kg (o.g.)	>1 year	n.a.	SIVmac251 (12 weekly doses 1 of AID50, i.r.)	65 d.p.i.	Yes (C-A DNA)	On-ART viremia in 4/4 RM (500–1000 copies SIV RNA/mL of plasma) at 0.3 mg/kg dose
	GS-986 or GS-9620	GS-986: 0.1 mg/kg; GS-9620: 0.05 or 0.15 mg/kg (o.g.)					Yes (C-A DNA)	On-ART viremia in 9/9 RM that peaked after 4th dose of GS-986; dose-dependent on-ART viremia levels
Del Prete et al. [69]	GS-9620	First course: 0.15 mg/kg (o.g.), 2 doses then 0.5 mg/kg, 10 doses; second course: 0.15 mg/kg (o.g.), 5 doses	75 weeks	n.a.	SIVmac239X (4 IU, i.v.)	13 d.p.i.	No (C-A DNA)	No on-ART viremia
Borducchi et al. [70]	GS-986	0.3 mg/kg (o.g.), 10 bi-weekly doses	50 weeks	Ad26/MVA therapeutic vaccine	SIVmac251 (500 TCID50, i.r.)	7 d.p.i.	No w/ GS-986 Yes w/ GS-986 + TV (C-A DNA)	No on-ART viremia
Borducchi et al. [71]	GS-9620	0.15 mg/kg (o.g.), 10 bi-weekly doses	96 weeks	PGT121	SHIV-SF162P3 (500 TCID50, i.r.)	7 d.p.i.	No w/ GS-9620 Yes w/ GS-9620 + PGT121 (C-A DNA)	No on-ART viremia
Bricker et al. [29]	GS-986	0.3 mg/kg (o.g.), 10 bi-weekly doses	40 weeks	Ad48/MVA therapeutic vaccine	SIVmac251 (10^5^ TCID50, oral)	28 d.p.i.	No (C-A DNA)	No difference in on-ART viremia between GS-986-treated RMs and controls

## Data Availability

Not applicable.
